# Motivation for weight loss among completers of a free community-based weight loss program in a US-Mexico border region: A self-determination theory perspective

**DOI:** 10.3389/fpubh.2022.652271

**Published:** 2022-09-20

**Authors:** Miriam Martinez, Cindy L. Salazar-Collier, Jessica Pena, Anna V. Wilkinson, Enmanuel A. Chavarria, Belinda M. Reininger

**Affiliations:** ^1^Texas Tech University Health Science Center, El Paso, TX, United States; ^2^College of Nursing & Health Sciences, Texas A&M International University, Laredo, TX, United States; ^3^School of Public Health, University of Texas Health Sciences Center at Houston, Houston, TX, United States; ^4^Rollins School of Public Health, Emory University, Atlanta, GA, United States

**Keywords:** motivation, self-determination, behavioral regulation, health promotion, obesity, weight loss

## Abstract

This study explores the perceptions and motivation for weight loss among participants who completed a free community-based weight loss program in a predominantly Hispanic and low-income region along the US-Mexico border using a Self-Determination Theory (SDT) perspective. This manuscript is timely as qualitative research on the effect of motivation as a factor in behavioral interventions to reduce overweight or obesity is currently lacking. Individual semi-structured interviews were conducted with 20 participants (80%, *n* = 16 female) who completed a community weight-loss intervention to assess motivation for weight loss and participating, and the role of social support and self-efficacy in weight loss. Directed content analysis was used with SDT guiding the questions and subsequent theme analysis. The findings communicate perspectives of participants relevant to 8 prominent themes. The regulation types and constructs related to SDT included: non-regulation, external regulation, introjected regulation, identified regulation, integrated regulation, and intrinsic regulation as well as competence and relatedness. Participants mentioned external sources of motivation, such as wanting to improve their physical appearance, and motivation due to financial incentives. Fewer participants reported intrinsic motivators, which the literature suggests are more likely to create lasting change and improved health behaviors. Understanding the motivation for behavior change and completion of weight loss programs is essential to help participants reach their goals effectively and sustain weight loss. A greater emphasis during weight loss programs on the motives for individuals to lose weight may help improve outcomes in weight-loss interventions. Additionally, increasing strategies targeted at enhancing intrinsic motivation for weight loss may be beneficial.

## Introduction

Obesity is an epidemic with a dire need for public health solutions (1–3). Efforts to decrease the rates of obesity have focused on interventions at multiple levels of influence including policy, environment, community and individual (4, 5). Although there are a variety of weight management interventions available, the success of participants is contingent on several factors including program completion, social support, and motivation for behavior change (6–8). It is important to study motivation at the individual level because motivation for weight loss has been shown to be a predictor for controlling weight successfully (6, 9, 10).

Motivation for diet and exercise has mostly been studied using quantitative approaches (11) such as the Treatment Self-Regulation Questionnaire (TSRQ) which assesses levels of motivation (12). A qualitative approach allows for further exploration of both motivational factors for weight loss (outcome) and associated behaviors (process) such as following a healthy diet or engaging regularly in physical activity. The investigation of the quality of motivation and its regulators can inform the understanding of effective weight loss efforts and interventions (13, 14). Further, theories such as the Self-Determination Theory expand on fundamental mechanisms for behavior change and may inform the development of more successful interventions (15).

Understanding motivational factors can help inform the improvement of weight loss programs especially as they pertain to an understudied population with an increased burden of overweight and obesity. A review of the literature revealed a dearth of information and published studies on motivational factors for weight loss among Hispanics, racial/ethnic minorities, and low-income populations. This is a problem as cultural attitudes and norms among minority racial and ethnic groups impact health behaviors in distinct ways and warrant study in their own right (16, 17). For example, Tamers et al. (18) completed a study assessing the relationship between worry of developing diseases associated with obesity and its role in motivating behavior change for physical activity and weight management among mostly Hispanic and non-Hispanic Black participants, with an average age of 44 years, high school education or less and living below the poverty line. Findings showed that individuals who were more concerned about the medical implications of being overweight or obese have a higher intention to change and are more likely to participate in health promotion programs (18). Research on the effect of motivation as a factor in behavioral interventions to reduce overweight or obesity is lacking (19, 20), particularly for Hispanics in lifestyle interventions (21).

Self-Determination Theory (SDT) was developed to inform social science and was first applied in the context of education and the effect of rewards systems on intrinsic motivation. Applications of SDT have since included physical and mental health outcomes, such as physical activity, tobacco cessation, medication adherence, weight loss, quality of life, depression, and anxiety (22). SDT has been applied to weight loss program for patients with morbid obesity (23) and showed that participants with greater autonomous self-regulation had increased reductions in their BMI as well as increased program attendance.

Other studies have explored the motivation for weight loss (6, 9, 24–27). Motivators for weight loss vary greatly across studies and may include health, physical appearance, social life, mood (24, 26, 27), desire to be a better parent/spouse, serving as a positive role model (19) and the desire to improve self-esteem and confidence through weight loss (26). Other weight loss motivations were identified in a latent class analysis conducted by Lemon et al. (19). Review articles have shown that predictors of successful weight control include self-motivation and internal motivation to lose weight (6, 9). On the other hand, an intervention conducted among rural adults of Nebraska found that while autonomous self-regulation was not a significant predictor of weight loss, controlled regulation was a significant predictor. While authors stated the finding could be partly explained by the provision of an incentive to program participants, findings still call to question whether intrinsic or extrinsic motivation is more significant and what this means for long-term sustainable change (28).

Reasons for weight loss may also vary by age and sex. The most extensive prospective study investigating successful weight loss maintenance among men in the National Weight Control Registry found that a health or a medical concern was the most common motivator for starting the weight loss journey (25). A web-based weight loss intervention among 301 women found that when physical appearance was a pre-intervention motivator it was actually associated with losing weight at a slower pace and gaining weight at 30-month post intervention (29).

A systematic review examining predictors of weight loss amongst participants of lifestyle interventions found predictors across four categories being sociodemographic, anthropometric, psychological/behavior, and intervention characteristic factors (30). Among psychological factors identified in studies of the review, physical appearance was found to be a significant predictor for women while autonomous self-regulation, self-efficacy, and social support were significant predictors of treatment outcomes among both sexes (30). As stated previously, there are conflicting results found across interventions, but not many of these studies have been conducted among predominantly underserved minority populations. Thus, this study aims to explore the perceptions and motivation for weight loss of participants who completed a free community-based weight loss program in a predominantly Hispanic and low-income region along the US-Mexico border.

## Methods

### Theoretical foundation of study

Self-determination theory (SDT) was used to frame the analysis for this qualitative study. The theory suggests that motivation, defined as psychological energy aimed at a specific goal, can be linked to autonomous or external influences. Autonomous regulation is internally driven and refers to behaviors originating from self. This may include core values and personal interests. In contrast, controlled regulation is externally driven and motivated by sources such as respect and admiration of others, monetary incentives, and favorable evaluations. Health behaviors are influenced by intrinsic and extrinsic motivation and overlap with three primary needs established by SDT: autonomy, competence, and relatedness. Autonomy refers to feeling in control of individuals' behavior. Competence involves the belief in individuals' skills, mastery, and ability to accomplish a particular task or action. Finally, relatedness is the need to feel a sense of belonging, connectedness with others, and social support (22).

**Figure 1:**
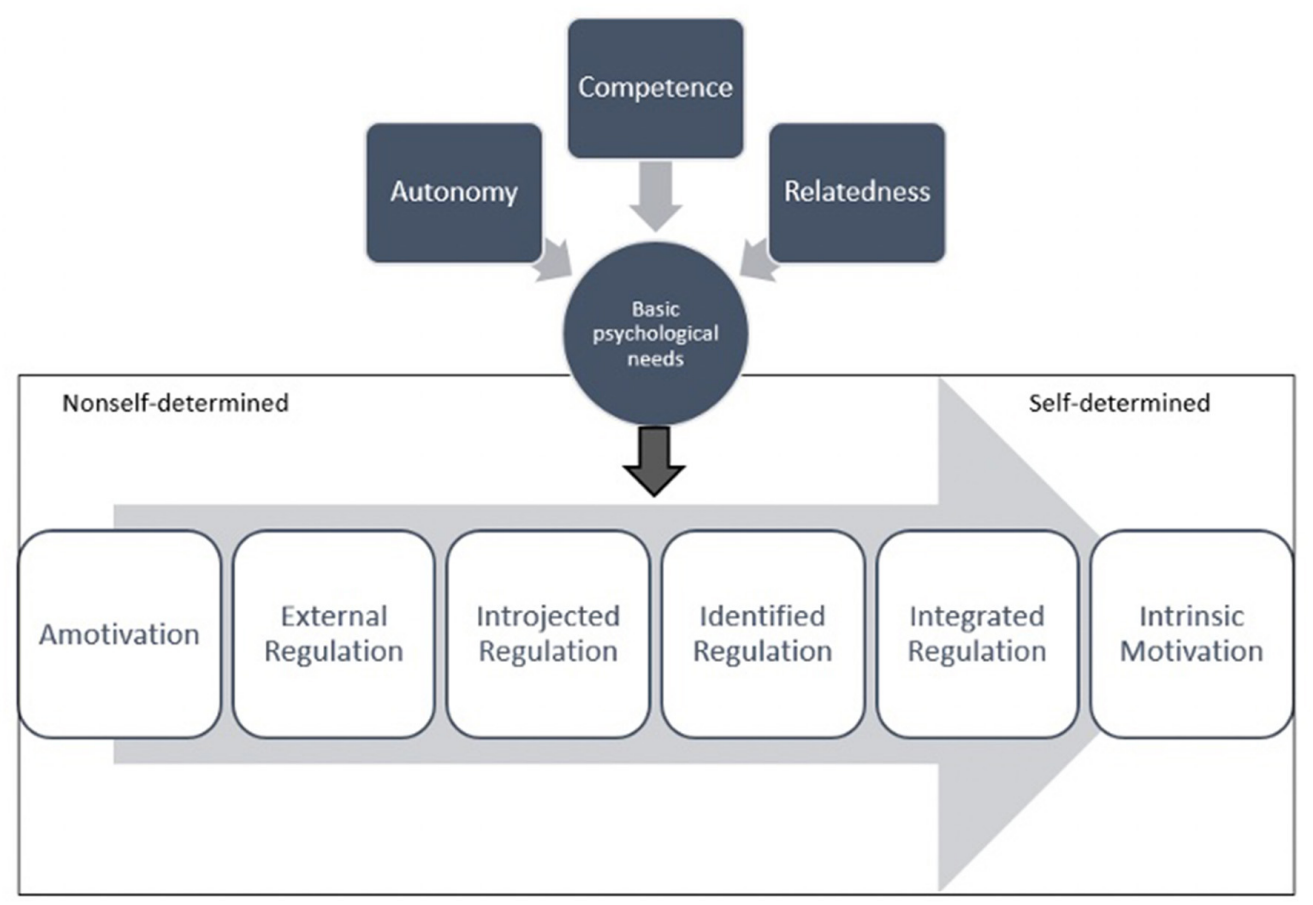
Self-determination theory psychological needs and the motivation spectrum.

The types of motivation seen in SDT are part of a continuum that can range from non-self-determined to self-determined. Further, there can be multiple types of motivation, driving a particular behavior. Along a continuum with non-self-determined motivation on the left of the continuum and self-determined motivation on the right, amotivation would present on the left with an impersonal source of motivation (Figure 1). The least internalized form of regulation is external regulation, which is engaging in a behavior to gain a reward or avoid a punishment. Introjected regulation is another type of extrinsic motivation that involves a response to prove something to oneself or others, or from feeling guilt or obligation to engage in a specific behavior.

Identified regulation, where an individual believes that a particular behavior is important to him/her, is further toward internalized regulation. Integrated regulation is closer to self-determined behavior along the motivation continuum. Integrated regulation is characterized by behaving in a manner that is consistent with personal values and other goals. Furthest right on the motivation continuum is intrinsic motivation, which is self-determined. This type of regulation is associated with personal interest, enjoyment, and inherent satisfaction in engaging in a particular behavior. It is important to note that in health behavior, forms of regulation are not always exclusive but rather may coexist within the same behavior and change over time and in different contexts (31, 32).

### Design and participants

A free community-based weight loss program has helped adults in the South Texas region become more aware of the benefits of weight loss through lifestyle changes related to improved dietary habits and physical activity. This three-month community-weight loss intervention titled, “The Challenge”, has demonstrated weight loss or maintenance in the majority of participants who complete the program (33). “The Challenge” is an annual voluntary program open to adults who are at least 18 years old, not pregnant, and free of medical conditions for which weight loss would be contraindicated. Participants are not required to be overweight or obese to participate. The registrations for “The Challenge” were held at community locations and worksites. Consent forms, participant information, and measures of adiposity were collected by trained staff at the beginning and end of each annual event. Participants were linked to free resources in the community, such as nutrition and exercise classes, and directed to local places to engage in physical activity. The program provided text message support and health coaching throughout “The Challenge.” Participants were encouraged to attend the final weigh-in, which was offered after approximately 14 weeks. Participants and teams with the highest percentage of weight loss received monetary prizes (33).

A total of 282 participants completed the 2019 Challenge and 93 participants were invited for an interview. Following the aim of this study, the sample included participants who attended the registration, and final weigh-in regardless of whether they met their weight loss goal. There were two phases of recruitment to ensure an adequate sample size. In phase 1 a table was set up by the researchers at one of the community sites (Site A) to recruit participants who completed “The Challenge.”

There were 53 participants who completed the final weigh-in at Site A. Participants at this site were invited to participate, and if they agreed, they provided their e-mail address and phone number to be contacted to set up an interview. From the 53 completers at the community site, 17 participants indicated interest and were contacted to schedule an interview. Eleven participants out of the 17 who had expressed interest in participating in the study were interviewed. In phase 2 of recruitment, e-mails were sent to a randomly selected group of 40 “completers” to invite them to participate in the study. For the participants recruited through e-mail, e-mails were sent to the potential participants to explain the research and invite them to join. Twelve of the 40 individuals e-mailed expressed interest in scheduling an interview. Of the twelve participants recruited through e-mail, nine were interviewed. Verbal consent was obtained to record interviews. A $20 incentive in the form of local grocery store gift cards was provided to participants. All aspects of this work received IRB and university approvals.

### Data collection

Individual semi-structured interviews were conducted with participants who completed a community weight-loss program in 2019 to assess motivational factors and their perception of determinants of their completion of “The Challenge.” Twenty participants completed interviews. The topic guide and probing questions were developed based on the Self-Determination Theory, which has been used to assess motivation for behavior change. Interviews were conducted in the participants' preferred language (English *n* = 17 or Spanish *n* = 3). Spanish transcripts were translated into English for analyses and disseminating findings. Interviews were audio-recorded and transcribed verbatim.

### Analysis

A directed content analysis approach was used for data analysis because it allows theory to guide the research question and subsequently identify constructs related to the theory (34). A deductive approach was used to elucidate motivation types and the constructs of competence and relatedness/social support from the participants' experiences. The motivation types related to the self-determination theory included: non-regulation, external regulation, introjected regulation, identified regulation, integrated regulation, and intrinsic regulation.

Transcripts were analyzed using ATLAS.ti 8 software. Coding and categorizing protocols were completed by two study team members with expertise utilizing SDT. Initially, transcripts were coded independently, then a comparison of code allocation followed. After initial independent coding, the coders met to discuss and reconcile codes for subsequent transcripts. Following this reconciliation, the two researchers individually coded the transcripts and met after every five interviews (4 meetings) to triangulate the data, enhance codes to more clearly address the research question, and examine emerging themes. Discrepancies in the codes were solved through discussion and consensus. At the final meeting, quotes that were most representative of the participants' experiences as they related to the SDT were selected. While there was an element of comparison that allowed for a discussion between the two coders a quantification of the degree of consensus was not calculated.

## Results

A total of 20 participants were interviewed between April and early June 2019, with interviews lasting between 20 to 50 min. The demographic characteristics of the interviewees are presented in Table 1. The majority of participants interviewed (80%, *n* = 16) were women and ranged in age from 28 to 60 years, with an average age of 41.5 (SD 10.5). Similarly, to the demographic makeup of the region, 95% of interviewees (*n* = 19) identified as Hispanic. Most of the participants had participated in “The Challenge” more than once (70%, *n* = 14), and an equal percentage were overweight and obese (45%, *n* = 9) at the beginning of “The Challenge.” Of the respondents, 95% lost weight and 30% (*n* = 6) were able to lose 5% or more of their initial weight. Average weight loss among all participants was 3.4 kg. Eight themes that were in accordance with SDT emerged from the participants' experiences. The themes were related to competence, relatedness, and six types of motivation represented in SDT (Table 2).

**Table 1:** Demographic characteristics of study participants (*N* = 20).

**Variables**		***n* (%)**
Age (years)		
	18–29	3 (15)
	30–39	6 (30)
	40–49	7 (35)
	50–59	3 (15)
	60–69	1 (5)
	≥70	0
Sex	Female Male	16 (80) 4 (20)
Ethnicity	Hispanic Non-Hispanic	19 (95) 1 (5)
Participation Category	Individual	8 (40)
	Small	9 (45)
	Large	3 (15)
Participated in previous “The Challenge” event	Yes	14 (70)
	No	6 (30)
Weight category/BMI at baseline	Normal	2 (10)
	Overweight	9 (45)
	Obese	9 (45)
Lost weight	Yes	19 (95)
	No	1 (5)
Average weight lost (kg)	3.4	
Weight Loss ≥5%	Yes	6 (30)
	No	14 (70)

**Table 2:** Identified themes per self-determination theory.

			**Amotivation**	**Extrinsic motivation**				**Intrinsic motivation**
**Theme**	**Competence**	**Relatedness**	**Non-regulation**	**External regulation**	**Introjected regulation**	**Identified regulation**	**Integrated regulation**	**Intrinsic regulation**
Description Example	Belief in the ability to meet weight loss goal	Support from others in losing weight	Lack of motivation or intention to lose weight	Weight loss to gain reward or avoid punishment	Weight loss to enhance worth or avoid guilt	Weight loss personally important or valued	Weight loss in harmony with values	Weight loss inherently satisfying

## Competence

Competence refers to the belief in one's ability to accomplish a goal or intended behavior change. “The Challenge” started in 2010, and participants can repeatedly enroll throughout the years. Of the participants interviewed, 70% had previously participated. Participants also had the option of participating in a small group of 2-10 people (45%, *n* = 9) or a large group of 11–20 people (15%, *n* = 3). Previous participation in “The Challenge” and being part of a group helped some individuals to feel more confident, “I was confident because not only had I done it before I knew I had a team to support me.” (Female, 42 years old).

Participation in “The Challenge” in prior years or previous experiences with weight loss could both boost and diminish competence based on the results of previous attempts. Not all of the participants who complete or participate in “The Challenge” or other weight loss attempts are able to meet their weight loss goals. Not being able to lose weight in previous years successfully became a source of doubt for some of the participants: “I didn't know if I was going to be able to lose weight because I've been going through several years that I have not been able to.” (Female, 51 years old). Thus, prior experiences could affect competence, both positively and negatively.

Along with free exercise and nutrition classes, “The Challenge” offers a Mid-Point Weigh-In along with a 5K run. This event serves to provide encouragement, a family-friendly activity, and an opportunity to assess progress. One participant shared how she was able to complete the 5K run.

I have this particular running song that helps me when I run a 5K, and I'm having a hard time. And I was in my head. Singing the song in my head. And I said, “I can do it. I can do it.” So, I ran it, and I felt such an accomplishment. To me, that was one of the motivators of “The Challenge.” (Female, 42 years old).

This participant used an affirmation rooted in her sense of competence. Earlier in her interview, she had mentioned that she was feeling ill but that she had been looking forward to the run, especially since she participated with her whole family, and her team was going to be there. She stated her goal was to at least walk, but after encouraging herself with affirmations of competence, she ran the entire 5K. In sum, there were various factors that influenced participants' sense of competence and included support from family and team members, participation in previous years, completion of milestones, and words of affirmation. These factors contribute to satisfying the need for competence, a core component of SDT.

## Relatedness

Relatedness refers to the need for social support, connectedness, and a sense of belonging. Participants found motivation through the support provided to them by their friends and families. Support was provided through verbal encouragement and providing accountability as well as through accompaniment in being physically active. One participant described, “My husband. He wouldn't let me quit. He would say, “you're going.” And my niece goes with me [to exercise] too, so that's another motivation.” (Female, 43 years old).

Participating in “The Challenge” as part of a group helped participants find encouragement and support to continue with their healthy behaviors. Having a group was especially important for participants during the more difficult times of their weight-loss journey. A participant describes the mutual support provided through her group, “When I had downfalls, my friends would pick me up. And when they had discouragement, I would pick them up. We would take turns, so it was very important for them to be part of the group.” (Female, 31 years old).

“The Challenge” and its pervasiveness in the city helped participants recognize that becoming healthier and losing weight is a common goal. For example, participants discussed how “The Challenge” brings many people together to lose weight, and that action was motivating to see.

I really like that everyone is on the same page. You see thousands of people registered trying to get healthy. And I think talking to these people and talking about how difficult it is for everyone and how it's not easy, everyone being on the same page is the biggest motivation. I think that's one of the coolest things. (Female, 28 years old).

A sense of connectedness through sharing a common goal can contribute to the need for relatedness. Other participants also talked about the “hype” of “The Challenge” and how it was exciting to see others also making an effort to improve their lives. Many of the participants expressed the importance of social support and relatedness in their weight loss attempts. The prevalence of this theme is consistent with the SDT identification of relatedness as an innate psychological need.

## Non-regulation

Non-regulation refers to the lack of motivation or intention to act. This program was voluntary, and only people who completed “The Challenge” participated in this study; thus, the frequency of expressed non-regulation is less than might be expected from the general population or from those that register for “The Challenge” but do not complete it. Only one interviewed participant expressed thoughts that are consistent with non-regulation. From the beginning he stated that he had felt a sense of obligation to participate, saying, “Ugh, my wife kind of pressured me to do it.” (Male, 28 years old).

He reported that following setbacks in team progress with weight loss and feeling that his team would not win the competition that it was no longer worthwhile to continue with the program, their behavioral change attempts, or their intention to lose weight.

On our team, we didn't lose any weight. I lost maybe 10 pounds, and then the rest of my team didn't lose anything. So I was just like you know what “screw it we're not going to win the Team Challenge so might as well stop.” (Male, 28 years old).

This same participant also mentioned that this sentiment was shared at his workplace among other team members. The participant's employer organized a smaller competition within the larger program. He mentioned that one of the other teams was performing much better and that the chances of his team winning were minimal, so this made him feel that it was not worth continuing to try to win through losing more weight.

Non-regulation was not a prevalent theme and was primarily attributed to feeling pressured to participate, setbacks in team progress, and the perception that winning the competition portion of “The Challenge” was unlikely.

## External regulation

Many participants mentioned an external source as their motivation for participating in the program and for wanting to lose weight and pursue a healthier lifestyle. External sources of motivation included: Improving physical appearance and receiving compliments, not disappointing others, and a desire to win the competition and prizes.

Participants were motivated not just by how losing weight made them look better; they also liked that others started to notice and compliment them on their physique. One participant put it as “I started looking thinner and looking better. People were saying, “Oh, you look good.” So that kind of kept me going. The results. So the results after 2 weeks. I kept going, and I was feeling stronger” (Female, 34 years old). Another participant mentioned that her main motive for wanting to lose weight, “Had to be cosmetic at first and I think especially because I was younger at the time. I was pretty young, and there weren't any health issues. Health was not the first thing on my mind, so it was all cosmetic. Trying to get into certain sizes, look a certain way.” (Female, 28 years old) This quote exemplifies wanting to lose weight to improve personal appearance and fit in with society's standards of beauty.

The group winners of “The Challenge” were determined by calculating the cumulative team progress. Participants expressed that they were motivated by their desire to be dependable and not let their teammates down. A participant who had previously participated as an individual indicated that it was an essential part of her success to be on a team because of the accountability it provided. She stated, “But this time around [entering “The Challenge”] because it was other coworkers, I didn't want to let them down either. And I didn't want to be the one who didn't lose any weight or didn't attempt to make a difference.” (Female, 44 years old).

There was a strong sense of being accountable to other members of the team. If you registered as part of a team, then it was important to take it seriously because your performance affected the group as a whole: “If you're part of a team you know like you're not just letting yourself down you're letting the team down. I think that's worse than you not doing it.” (Male, 28 years old) Thus the perception of the importance of dependability and accountability was a motivation source for participants.

“The Challenge” also had an incentive component. The participants that had the highest weight loss percentage qualified for cash prizes. Some participants mentioned that their motivation was winning the competition, “We are in it to win it. We didn't win it, but that was our motivation” (Female, 44 years old). Another participant mentioned that she was excited at the prospect of winning prizes.

“I really like the way they do “The Challenge.” The hype they put into it. It's just really exciting. I just love the whole hype. You know how they promoted it and everything. The prizes are very exciting” (Female, 42 years old).

Gift cards were awarded to everybody who reported a 5% body weight loss from their initial body weight or who had been involved in program activities such as exercise classes. Involvement in activities was measured using stickers that were provided by partnering gyms and organizations. Other participants found it motivating to see the progress they were making through the stickers they had collected.

“Those stickers kind of reminded me of how many classes I had been to, where my journey started and where I was heading. So physically seeing the sticker, even if I wasn't winning anything. Just knowing that little sticker was making sense, that every day I put something on that sheet. I was doing something. It kind of affirmed what I was doing for me.” (Female, 34 years old).

External motivators were highly cited as influencers to be involved in “The Challenge.” Participants mentioned their desire to improve their physical appearance and the associated compliments, the importance of accountability from their team, and the prizes associated with successfully losing weight. External motivators are one component of motivation in SDT and the extent and depth of comments around this influencer suggest this is a primary motivation for the participants in this study related to weight loss and completion of “The Challenge.”

## Introjected regulation

Introjected regulation is rooted in enhancing self-worth or avoiding guilt. Improving self-worth was consistent with mental health benefits and improved self-esteem. Participants found that after exercising and eating well, they also started to feel better about themselves, thus positively contributing toward their mental health. A participant who began regularly exercising during “The Challenge” stated,

“I noticed that when I eat well and when I work out, I feel good…a lot of it has to do with self-esteem, like physically, emotionally when I'm eating clean and when I work out, I feel good, and my clothes fit better, and that just makes me feel more at peace with myself.” (Female, 28 years old).

In contrast, a few participants were more concerned with avoiding guilt as one participant stated she would, “feel guilty on days that I didn't exercise” (Female, 44 years old).

Another participant said that she would tell herself, “Okay, you gotta do this, cause you can't go back. You'll look […] that you didn't lose anything, or you gained.” (Female, 34 years old) So, while participants were aiming to improve their self-esteem, others were acting to avoid more negative self-talk and feeling guilty due to not exercising or losing weight. Introjected regulation and the internalization of improving self-esteem and avoiding guilt as mentioned by participants in this study, is included in the continuum of behavioral regulations and is closer to more self-determined behavior than external regulation.

## Identified regulation

Identified regulation is behaving in a manner that is consistent with personal values and other goals. Participants found personal value and importance in engaging in healthy behaviors. Participants mentioned that they were engaging in behavior changes not only for the duration of The Challenge but for the rest of their lives. A participant expressed, “One of the motivations was that I was getting results and he [husband] was getting results. So that's an incentive… and we see it short term for The Challenge, but really this is our way of life.” (Female, 57 years old).

One participant mentioned that “After the Challenge is done, there is nothing you are going to win but be healthy” (Female, 44 years old). Another participant elaborated on choosing to participate in The Challenge as something that she values and does for herself. She said, “When I did this Challenge, one of my goals was to eat healthier for myself and to exercise on a regular basis for myself” (Female, 42 years old). These expressions demonstrate identified regulation and that participants find it worthwhile and valuable to engage in healthy behaviors. In congruence with SDT this type of regulation is a more self-determined form of motivation and can be a positive regulator for participants in a weight loss program such as “The Challenge.” Identified regulation has been associated with increased enjoyment and interest in a behavior and a desire to expend more effort to accomplish a goal.

### Integrated regulation

This type of motivation demonstrates how behavior changes to achieve a healthier life can be in harmony with other values and personal goals, such as taking responsibility for one's health, avoiding adverse health outcomes and being around for their family.

Participants were motivated to make lifestyle changes based on health advice from their doctors' visits. The physician that this participant describes demonstrates respect for participants' need for autonomy by providing suggestions but leaving the participant to make the decision about what course to take to prevent further elevation of his blood pressure.

I had gone to the doctor last year. And he says, look, man, your blood pressure is kind of getting high. You're getting really borderline. We can do one of two things: we can give you medication for diabetes and blood pressure, or you can change your lifestyle. Change your diet. However you want to do it. It's up to you. And basically, I said I'm going to change my lifestyle. (Male, 49 years old).

A few participants mentioned that the reason that they participate in “The Challenge” is due to concern about developing diseases commonly found among their families. One participant said that she has been making positive progress in controlling her blood sugar and lost two of her siblings to complications from diabetes. “I've been seeing the progress that I've been making, and my A1c right now is at 5.6 so, it's good, but I have to make that change because you know family history is really bad…I lost two brothers who were on dialysis.” (Female, 58 years old) Thus, wanting to slow or halt the progression of poor health outcomes serves as a motivation for participants of “The Challenge.”

The motivation in some cases was to be able to live long and well enough to meet their grandchildren and to be around for other important moments in their families' lives. One participant was looking forward to the birth of her grandchildren. She said,

“I'm also going to be a grandma in 2 weeks. So, I thought this was the best opportunity to get healthy. Because I want to be around longer because now I have grandkids coming into the world. That's a big reason to motivate me now to be healthier.” (Female, 44 years old).

Achieving a healthier life is important for other participants because it could potentially allow them to bear witness to the marriages and graduation of their children. There were references made to preventing adverse health outcomes such as amputations and blindness, but this was mostly in the context of avoiding these medical problems to be able to enjoy their families longer and more fully. One participant stated,

I want to see my children graduate from college and hopefully get married. I want to be around for them when I'm older, and that's not going to happen if I go back to my [unhealthy] lifestyle. If I go back, I'm going to die young. I'm not going to see 60 or 70 or whatever. Or I'm going to be amputated with a leg or whatever or lose my eyesight. (Male, 49 years old).

In some cases, it was essential to engage in healthy habits to improve the health of their entire family. The value that was emphasized in this next quote was caring for her family and changing the habits of her children as well. This mother shared, “I wanted to include my children in my healthy habits. This year, I wanted not only to eat healthy for myself but to cook healthier meals for my kids, to limit their sugars and to include more exercise in their routine.” (Female 42 years old) This demonstrates integrated regulation in that the participant valued taking care of her family and fostering a healthy environment for her children. Her behavior change was in harmony with her serving as a role model and providing her children with nutritious meals and an active life. Integrated regulation is closer to self-determined behavior along the motivation continuum and is characterized by behavior that is consistent with personal values and other goals including taking control of health outcomes, avoiding negative health effects, being present for family, and setting good examples.

### Intrinsic regulation

This type of motivation refers to behaviors and activities that are described as enjoyable or inherently satisfying. Intrinsic regulation can be seen when participants mentioned that they became physically active because it became something that they enjoyed doing. For some, it provided a pleasant time with their loved ones. This woman described going out with her husband, “We go hiking every morning and then every evening, we just do stuff. And if there's other parks nearby, we go to the other parks. That kind of stuff, and we enjoy ourselves very much” (Female, 57 years old). She mentioned in her interview that she and her husband were able to enjoy nature and the scenic areas when they went on their hikes.

Several participants expressed that engaging in physical activity became something that their family started doing together for fun.

You know it definitely helped that he [husband] came with us… we went on walks on the weekends or bike rides, and he would come with us. It was encouraging to have it become a family fun thing. That's what it became, something to do as a family for fun. (Female, 42 years old).

Another participant expressed that she was “very scared about the exercise part of it, and it ended up being the easiest thing for me because I found something that I actually enjoy” (Female, 28 years old). In this case, it was no longer just exercising as part of creating a calorie deficit for weight loss but rather something that she found fulfilling because it was enjoyable.

## Discussion

The themes found in this qualitative analysis elucidated how the Self-Determination Theory is reflected in the experiences of participants who completed a free community weight-loss program. The premise of this theory is that autonomy, competence, and relatedness are psychological needs and influence intrinsic and extrinsic motivation for behavior. The types of motivation are part of a continuum that can range from non-self-determined to self-determined.

In line with SDT, relatedness is a psychological need that influences motivation and can facilitate the adoption of health behaviors. This program had built-in social support provided through community events, group participation, social media, and text messaging. Social support and relatedness was an important component of the weight loss and behavior change experience for many of the participants. This is consistent with previous literature that demonstrates group support is conducive to weight loss (35, 36). Participants mentioned that they felt supported by the members in their team and that they would provide encouragement to one another. Other participants expanded on the social support offered by their family members. While social support can be beneficial, loss of self-determination, such as continued pushing by a spouse or significant other to lose weight, may negatively influence the internalization of weight loss as an autonomous health goal (37). For the 43-year-old female participant that stated that her husband “wouldn't let me quit,” she said that she found his support to be helpful. The 28-year-old male that mentioned his wife had pushed him into registering for “The Challenge” had more expressions consistent with amotivation and, thus, less behavior internalization and self-determination.

Our study found many similarities with what has previously been described in the literature regarding reasons for weight loss motivation. Similarly to other studies, motivators identified in this study included both extrinsic and intrinsic motivators such as (1) the prospect of winning financial incentives provided by weight-loss interventions (38, 39) (2) striving for social approval and avoiding feelings of shame (3) physical appearance, (4) health outcomes, and (5) valuing health and healthy behaviors (24, 26). SDT showcases that the forms of regulation and intrinsic and extrinsic motivation are not mutually exclusive but rather may coexist within the same behavior and change over time and in different contexts (31, 32). The motivation for behavior reflected in the experiences of participants in this study displays the continuum and overlap from non-self-determined to self-determined behavior.

Participants in this study endorsed financial incentives as a source of motivation. Evidence supports that weight loss outcomes are improved with the addition of financial rewards (38, 39). In an internet intervention modeled after Diabetes Prevention Program examining the treatment of obesity in lower-income women, women who also received small financial incentives for self-monitoring and losing weight, lost approximately three times more weight, than those completing the program alone. This shows that modest financial incentives can improve weight loss in women from financially disadvantaged backgrounds (40). Participants also mentioned they were motivated by the prizes and financial incentives offered as part of “The Challenge.”

Behavior change motivated by external factors such as feelings of guilt or disappointing others is not self-determined and is more prone to dissuasion following negative experiences and perceived failures (41). Other studies have found that striving for social approval and avoiding feelings of shame do not help improve healthy eating behaviors, such as increasing fruit and vegetable consumption and reducing intake of foods with added sugar. Rather than improving eating behaviors, it is more likely that these motivators place individuals at a higher likelihood of engaging in risky practices, such as intense fasting, to control weight (42). Only a few participants mentioned that they felt guilty when they did not exercise. Avoiding guilt is a component of introjected regulation and can be considered positive if it fuels an individual to engage in a healthy behavior such as exercising, however it can negatively affect perceived wellbeing from not following social expectations (42).

Wanting to improve physical attractiveness was another motivator for participants. Societal standards of attractiveness include thinness for women and increased muscularity for men (43). Attempts to conform to these standards through weight loss is seen more often in women with an increased motivation to exercise to manage weight, and enhance their attractiveness (44). Among the four men interviewed, there was no mention of wanting to lose weight or participate in “The Challenge” to increase their physical appearance. Female participants in this study who mentioned gaining confidence in their appearance also cited improved mental health benefits. The dual nature of the benefits reflected the introjected and intrinsic regulation constructs. The motivation to conform to standards of attractiveness is externally regulated and consequently has a lower degree of self-determination whereas improved confidence from weight loss is a potential value closer to intrinsic motivation.

Results from our study are consistent with previous findings about motivators for weight loss that include worries about health conditions (45). Participants mentioned that they wanted to improve their health, secondary to their worry about the adverse consequences of an unhealthy life. Participants reported worry about blindness, amputations, early death, and dialysis.

Individuals who feel susceptible to diseases commonly attributed to obesity may be more likely to exercise and maintain a healthy weight (46). This is in accordance with the magnitude of worry or concern about disease susceptibility and the link to perceived risk (47, 48). Worry and concern about susceptibility can have a positive effect through motivating individuals to engage in behaviors protective of their health (49). Although this is not an explicit construct of the SDT, perceived risk is a core component of other health behavior theories, including the Health Belief Model and Protection Motivation Theory. In our study, we found that health worry and concern were a major theme in the desire to lose weight and adopt healthy behaviors. The desire to maintain health and avoid health complications to spend more time with family and enjoy the milestones in their families' lives, such as graduations and weddings, was a repeated theme throughout. This is consistent with integrated regulation where the goal of maintaining health was in harmony with valuing time with family.

Participants reported valuing health and healthy behaviors which are more toward the continuum of self-determined behavior and intrinsic motivation. Previous research has shown that engagement in behaviors that are inherently satisfying and autonomously regulated are positively associated with more significant weight loss and improved weight loss maintenance (50, 51). In this study participants reported that they enjoyed engaging in physical activity which is in line with the development of intrinsic motivation.

A significant source of motivation, and link with SDT that has not been previously documented in the literature, is the multiple ways in which family affects motivational regulation in this predominantly Hispanic population. Participants mentioned not wanting to suffer from health conditions that had affected their family members. Motivators included wanting to improve health to be around for important family events as well as the value of providing a healthy environment and example for their children. Finally, family was also present in intrinsic regulation as participants mentioned that they ended up finding physical activity to be enjoyable because it was something that they could do together as a family. This emphasis on family is consistent with the strong orientation toward family, or familism (52). Although this value is found in other cultures it is very relevant among Hispanics cultures (53). Studies have shown that involving families and incorporating lifestyle changes that can be implemented at home are effective at helping Mexican Americans lose weight (54). Overall, in this study family is a consistent motivator present in different regulation types outlined by SDT.

### Strengths and limitations

A strength of this study is the rich narrative provided by participants that allow for the expansion of studies focusing on measuring and characterizing motivation quantitatively. This study also had a predominantly Hispanic population. Researcher triangulation helped to ensure that the quotes selected were representative of the themes and constructs found in SDT. Limitations include sample selection, social desirability, and researcher bias.

The study sample was comprised of participants who completed a weight loss program for which they self-enrolled. Considering that not only did interviewees sign-up up for the program but also completed it and the majority lost weight, it could be said that there was a strong sense of motivation among this group of participants, thus contributing to selection bias. The findings may not be as generalizable as they would have been if non-completers of “The Challenge” had also been interviewed. It would be helpful in future studies to interview participants at the beginning of “The Challenge” and stratify participants in analyses by completion status. This would help to determine the characteristics of the SDT in this population that may be more conducive to completing “The Challenge” and accomplishing weight loss.

Given that participants were interviewed by the researchers, they may be more inclined to provide responses that they may perceive as desirable. Researcher bias may have also influenced information provided by the participants and interpretation of this data. The biases mentioned above are inherent in this type of research and can result from the questions that are asked, how data is analyzed, coded, and interpreted. Bias was also reduced by triangulating data.

## Conclusion

This study explored the perceptions and motivation for weight loss of participants who completed a free community-based weight loss program in a predominantly Hispanic and low-income region along the US-Mexico border. Many participants mentioned external sources of motivation, such as preventing adverse health outcomes, wanting to improve their physical appearance, and being motivated by financial incentives. Fewer participants mentioned intrinsic motivators, which are more likely to create lasting change and improved health behaviors. Understanding the motivation for behavior change and completion of weight loss programs is essential to help participants reach their goals effectively. A greater understanding of the motivations for individuals to lose weight may help improve outcomes in weight-loss interventions. Additionally, increasing strategies targeted at improving intrinsic motivation for weight loss may be beneficial.

## Data availability statement

The raw data supporting the conclusions of this article will be made available by the authors, without undue reservation.

## Ethics statement

The studies involving human participants were reviewed and approved by UTHealth Committee for the Protection of Human Subjects. The patients/participants provided their written informed consent to participate in this study.

## Author contributions

The writing and first draft of the manuscript was led by MM. MM and CS-C completed data analyses and JP helped with the topic guide, data collection and review of the manuscript. AW, EC, and BR provided critical revisions and EC and BR provided conceptual guidance throughout this project. All authors contributed to the article and approved the submitted version.

## Funding

Funding for The Challenge comes in part from the City of Brownsville, local sponsors and UTHealth. This study was partially supported by the Center for Clinical and Translational Sciences, which is funded by National Institutes of Health Clinical and Translational Award no. UL1 TR003167 from the National Center for Advancing Translational Sciences. The content is solely the responsibility of the authors and does not necessarily represent the official views of the National Center for Advancing Translational Sciences or the National Institutes of Health. Funding for analysis software and transcription services were provided by the University of Texas Rio Grande Valley School of Medicine Office of the Associate Dean of Research.

## Conflict of interest

The authors declare that the research was conducted in the absence of any commercial or financial relationships that could be construed as a potential conflict of interest.

## Publisher's note

All claims expressed in this article are solely those of the authors and do not necessarily represent those of their affiliated organizations, or those of the publisher, the editors and the reviewers. Any product that may be evaluated in this article, or claim that may be made by its manufacturer, is not guaranteed or endorsed by the publisher.
